# Melatonin a day: Mitigates saline-alkali stress away!

**DOI:** 10.1093/plcell/koaf039

**Published:** 2025-02-21

**Authors:** Meenu Singla-Rastogi

**Affiliations:** Assistant Features Editor, The Plant Cell, American Society of Plant Biologists; Department of Biology, Indiana University, Bloomington, IN 47405, USA

By 2050, global warming is expected to significantly worsen drought conditions and disrupt rainfall patterns, leading to critical freshwater shortages in many regions worldwide. Soil salinization is also a growing problem in agricultural lands, primarily due to human activities and climate change. Over one-half of the world's arable land faces the dual threats of salinization and desertification (Tarolli et al. 2024). According to a recent report by the Food and Agriculture Organization of the United Nations, more than 1.4 billion hectares of land are affected by salinity, alkalinity, or a combination of both, causing severe abiotic stress that hinders agricultural productivity, especially in areas with a scarcity of freshwater. Plants growing in saline-alkali soils are exposed to a range of detrimental factors, including elevated levels of sodium ions (Na+), high pH, and osmotic stress (Zhao et al. 2020; Zhang et al. 2023). In such conditions, plants experience redox imbalances, primarily due to the accumulation of reactive oxygen species and reactive nitrogen species. These harmful compounds disrupt normal cellular functions and contribute to oxidative and nitrosative stresses, respectively (Sies et al. 2024), both of which impair plant growth, reduce crop yields, and compromise overall plant health. Therefore, understanding the mechanisms underlying saline-alkali tolerance is crucial for advancing agricultural productivity in regions grappling with soil salinity and alkalinity.

New work by **Jin-Wei Wei, Minghui Liu, Dan Zhao, and colleagues (**Wei et al. 2025**)** offers critical insights into how tomato (*Solanum lycopersicum* L.) plants respond to saline-alkali stress. The researchers highlight the roles of melatonin and nitric oxide (NO), a common reactive nitrogen species, in modulating the plant's reaction to saline-alkali stress (Figure). NO, well-known for its role as a signaling molecule, can induce nitrosative damage by modifying various biomacromolecules via S-nitrosylation. A key product generated through this process is S-nitrosoglutathione (GSNO), which functions both as a stable intracellular reservoir of NO and a transport vehicle for NO across cells (Fancy et al. 2017). The cellular concentration of NO is tightly regulated by the enzyme GSNO reductase (GSNOR), which catalyzes the degradation of GSNO, thereby controlling NO levels (Lee et al. 2008; Gong et al. 2019; Qin et al. 2023).

**Figure.**
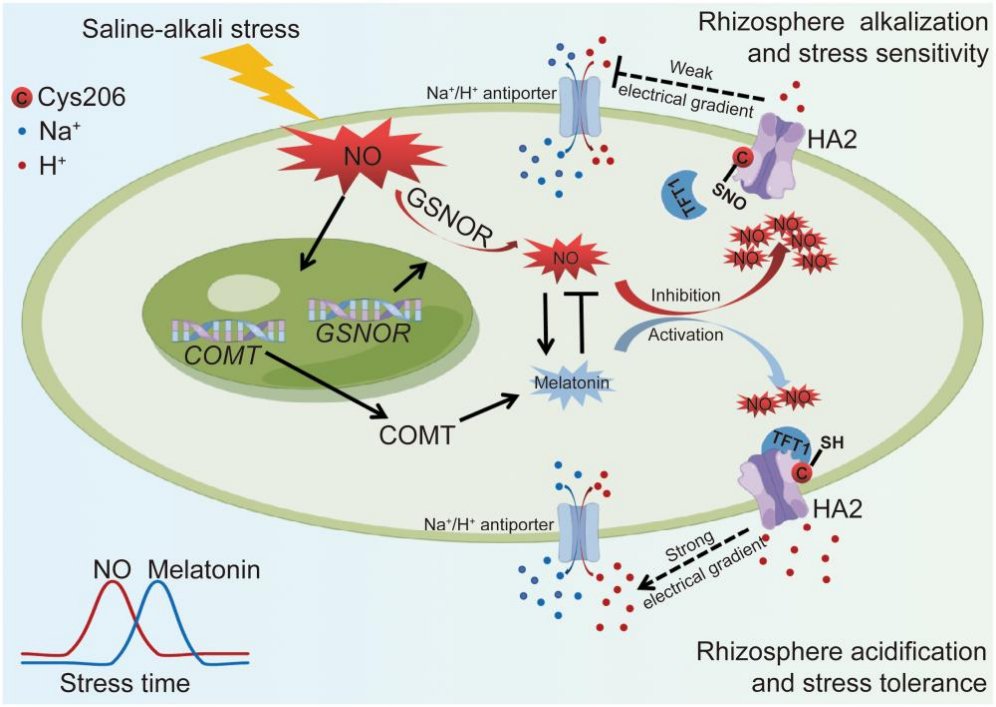
A model for melatonin's role in plant response to saline-alkali stress. In saline-alkali stress, excess NO accumulates in tomato roots, resulting in nitrosative damage. NO is degraded by GSNOR. Conversely, NO stimulates COMT transcript and melatonin synthesis to scavenge NO. Therefore, melatonin alleviates nitrosative damage, especially proteome S-nitrosylation, caused by stress-triggered NO. Based on the well-known physiological functions of HA, this study highlights that HA2 is S-nitrosylated at Cys206 in long-term saline-alkali stress, reducing HA activity, H+ efflux, and saline-alkali tolerance by impairing the HA2-TFT1 interaction. If COMT-mediated melatonin scavenges NO in time, it can effectively alleviate the HA2 S-nitrosylation to promote HA2-TFT1 interaction, resulting in enhanced HA activity, H+ efflux, and saline-alkali tolerance. Therefore, melatonin and NO are proposed as a pair of redox switches to control tomato saline-alkali tolerance by regulating HA2 S-nitrosylation at Cys206. Reprinted from Wei et al. (2025), Figure 10.

First, Wei et al. (2025) demonstrated that saline-alkali stress induces a surge of NO, resulting in widespread nitrosative damage across the entire proteome through extensive S-nitrosylation. Interestingly, they show that this damage is mitigated by GSNOR activity but only during the early phase of stress induction, prompting further investigation into additional mechanisms by which plants can scavenge NO and reduce its deleterious effects in the long term. Next, the authors investigated a non-enzymatic melatonin-mediated NO scavenging pathway. Melatonin, known for its role as a neurotransmitter and stress modulator in animals, has recently garnered attention in plant biology for its diverse roles in regulating plant growth, development, and stress responses (Sun et al. 2020; Arora et al. 2024). In plants, melatonin is synthesized via 6 key enzymes, with caffeic acid O-methyltransferase (COMT) gene family member acting as the rate-limiting enzyme in this pathway (Back et al. 2016). Previous studies have shown that overexpression of COMT enhances melatonin production in plants, which, in turn, improves stress tolerance (Sun et al. 2020).

Wei and colleagues analyzed the effects of overexpressing or knocking down COMT and GSNOR in tomato plants. Their findings reveal that GSNOR promotes saline-alkali tolerance by mitigating nitrosative damage. Simultaneously, COMT enhances melatonin synthesis, facilitates NO scavenging, and improves overall stress tolerance. Notably, this work sheds light on the time-dependent interaction between melatonin and NO, especially in the context of melatonin's influence on redox-sensitive processes, such as S-nitrosylation across the proteome. Using fluorous affinity tag switch-based quantitative S-nitosoproteomics (Qin et al. 2023), the authors identified specific S-nitrosylated proteins responsive to saline-alkali stress. One significant discovery from this work is the impact of S-nitrosylation on plasma membrane H+-ATPase 2 (HA2), an essential enzyme for maintaining cellular pH and ion homeostasis. The authors demonstrated that S-nitrosylation of HA2 impairs its interaction with 14-3-3 protein 1 (TFT1), thereby disrupting its proton (H+) efflux activity and diminishing the plant's ability to cope with saline-alkali stress. Interestingly, they further confirmed that COMT-mediated melatonin production counteracts this modification by preventing the S-nitrosylation of HA2, restoring its functionality, and improving the plant's tolerance to saline-alkali stress. The authors then generated a nitrosylation null version of HA2, which was insensitive to the effects of NO.

Building on these findings, the authors propose a model wherein melatonin and NO act as redox switches, modulating S-nitrosylation to alleviate nitrosative damage and enhance plant stress resilience. Furthermore, the authors illustrated a translational impact of their discovery through grafting tomato scions onto rootstocks overexpressing COMT, GSNOR, or HA2, or genetically modifying the critical S-nitrosylation site (Cys206) of HA2, which can enhance both saline-alkali tolerance and overall productivity.

This study presents an overview on the “melatonin-NO-HA2” module and its role in mitigating nitrosative damage under saline-alkali stress conditions. Given the vital roles of melatonin, NO, and H+-ATPase in plant growth, yield, and stress tolerance, the next crucial step is to explore how this mechanism of cellular NO scavenging can be applied to enhance crop resilience in marginal lands affected by saline-alkali soils and promote sustainable agricultural productivity. Additionally, investigation of other nitrosylated proteins identified in this study, which may contribute to saline-alkaline sensitivity, is warranted.

## Data Availability

No new data were generated or analyzed in support of this research.

## Dive Curated Terms

The following phenotypic, genotypic, and functional terms are of significance to the work described in this paper:

GSNO CHEBI: CHEBI:50091

melatonin CHEBI: CHEBI:16796

GSNOR Gramene: AT5G43940
GSNOR Araport: AT5G43940

HA2 Gramene: AT4G30190
HA2 Araport: AT4G30190
